# Immunotherapy: Advancing glioblastoma treatment—A narrative review of scientific studies

**DOI:** 10.1002/cnr2.1947

**Published:** 2023-12-09

**Authors:** Sagun Tiwari, Zhenxiang Han

**Affiliations:** ^1^ Net Fresh Hospital Chitwan Nepal; ^2^ Shenzhen Key Laboratory of Immunomodulation for Neurological Diseases Shenzhen Institute of Advanced Technology, Chinese Academy of Sciences Shenzhen China; ^3^ University of Chinese Academy of Sciences Beijing China; ^4^ Department of Neurology and Rehabilitation Seventh People's Hospital of Shanghai University of TCM Shanghai China

**Keywords:** cancer, clinical studies, glioblastoma, glioma, immune checkpoint, immunotherapy, microbe, microbiome, tumor

## Abstract

**Background:**

Glioblastoma (GB) is an aggressive and deadly brain tumor with a poor prognosis despite the current standard of care, including surgery, radiation, and chemotherapy.

**Recent Findings:**

In recent years, there has been increasing interest in the potential of immunotherapies, seen to be effective in treating other cancers, in the treatment of GB. This comprehensive review presents an in‐depth analysis of the remarkable progress of immunotherapy in GB treatment, focusing on human clinical studies. It also analyzes the current findings, challenges, and limitations that underscore the transformative potential of immunotherapy in managing GB. Of particular significance, it delves into the intriguing interaction of the human microbiome with immunotherapy as a novel avenue for enhancing treatment outcomes of GB.

**Conclusion:**

This study sheds light on the complex GB therapy landscape and the cutting‐edge strategies that show promise for enhancing patient prognosis.

## 1 BACKGROUND

Glioblastoma (GB), an aggressive and deadly brain tumor of the central nervous system (CNS) (WHO grade IV astrocytoma), affects less than 10 per 100 000 persons globally.^1^ Despite advancements in medical and scientific technology, the standard of care procedures for GB patients, including surgical tumor resection, radiotherapy, and chemotherapy with temozolomide, has led to a short survival time of approximately 1.2 years after primary diagnosis with adverse events.

In search of effective and reliable treatment, researchers and healthcare professionals have turned to the promising field of immunotherapy, which aims to harness the immune system's ability to detect and eradicate cancer cells. This treatment approach has gained significant attention in recent years with the approval of drugs such as Ipilimumab, Sipuleucel‐T for cancers.^2^, ^3^ However, the aggressive pace of growth, ability to infiltrate brain tissue, molecular heterogeneity, and resistance to the treatment make GB particularly dangerous. Despite the promise of immunotherapy, targeting the immune mechanism in the CNS remains challenging because of GB's immunosuppressive environment. To tackle this challenge, it is essential to overcome intrinsic resistance, address systemic immunosuppression, combat adaptive resistance, and adapt to acquired resistance. Immunotherapy also faces several other limitations, including the presence of the blood–brain barrier, variations in patient response to treatment, and a moderate to high grade of adverse events.^4^ Several vital elements significantly impact the restricted immune response inside the central nervous system (CNS) milieu. First, a significant obstacle to the onset of adaptive immunity is the lack of professional antigen‐presenting cells (APCs) in the CNS parenchyma. Specialized APCs play a crucial role in immunological activation by digesting and presenting antigens to lymphocytes, such as dendritic cells (DCs). Major histocompatibility complex (MHC) class I and II molecules, essential for antigen presentation, are also not highly expressed in the CNS. This downregulation of MHC molecules may hamper T cell recognition of CNS‐derived antigens. Furthermore, the lack of traditional CNS lymphatic drainage further restricts the immune system's capacity to efficiently monitor and react to antigens within the CNS. Together, these elements create a distinct immunosuppressive milieu inside the CNS, which presents difficulties for the immune system's development against antigens derived from the CNS and emphasizes the complex relationship between immunological privilege and neuroinflammatory processes in this particular niche.

The most considerable difficulty is overcoming the severe immunosuppressive milieu that GB creates in the brain. To change this milieu and allow the immune system to respond effectively, novel tactics are needed. Furthermore, it is crucial to comprehend and deal with the sophisticated defense mechanisms that GB uses to resist immunotherapies. Understanding the molecular and cellular causes of resistance can help with the creation of more resilient therapeutic approaches. Researchers have been exploring additional approaches, such as the potential integration of fasting with immunotherapy, to overcome some of these limitations and enhance immunotherapy's efficacy.^5^ A steadily increasing amount of research indicates that fasting along with other treatment options, may significantly contribute to the conditions that prevent the cancer cells from adapting and surviving, thereby improving the immune system's response to cancer cells.^5^

**FIGURE 1:**
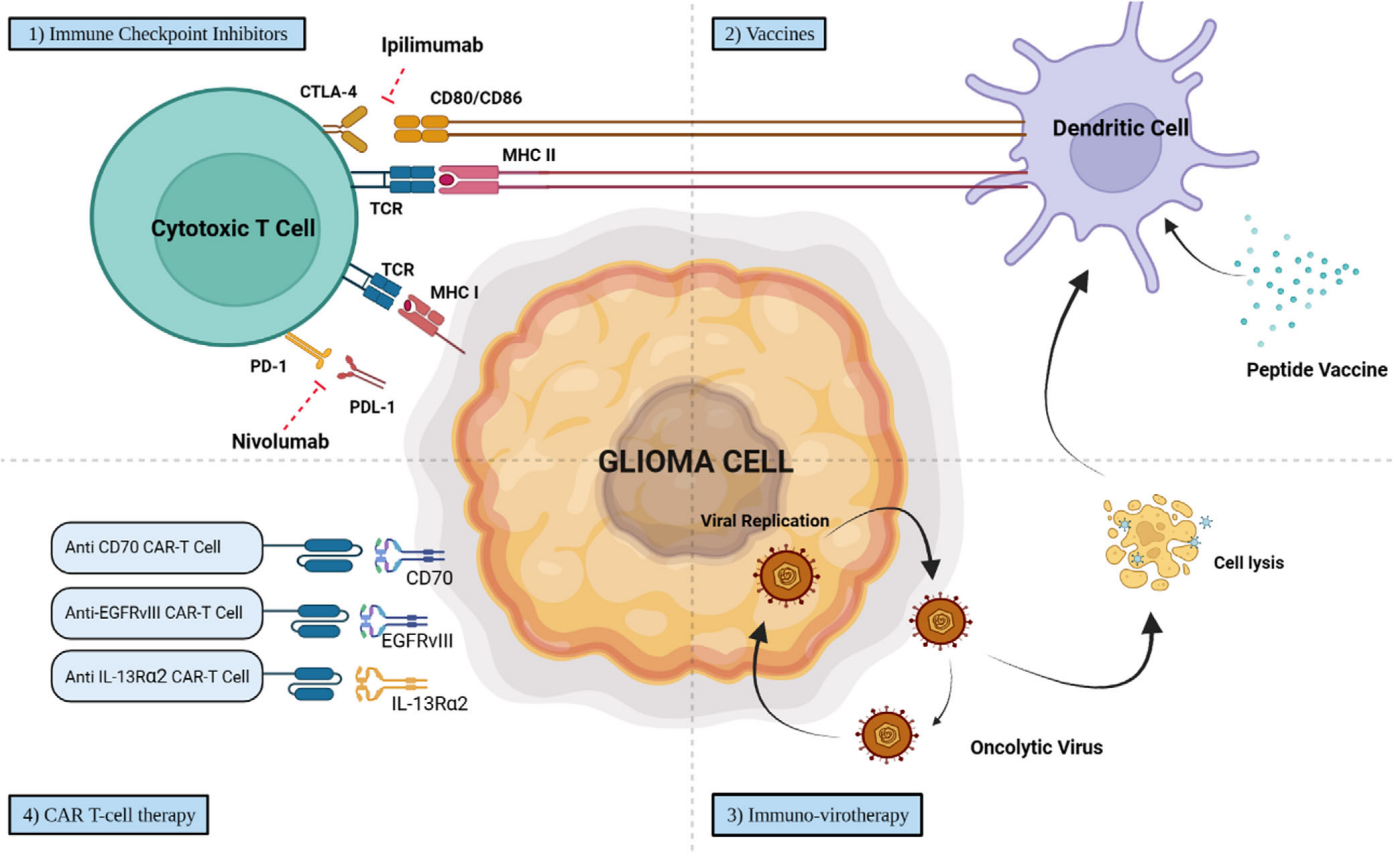
Commonly used immunotherapeutic approach to treat glioblastoma.

Despite these challenges, many clinical trials and immunotherapy studies for GB are ongoing, with some promising results reported. To examine the field's current state and evaluate immunotherapy's effectiveness in treating GB, this review analyses the latest innovative immunotherapeutic approaches, including immune checkpoints, vaccines, oncolytic immune‐virotherapy, and CAR T‐cell therapy (Figure 1 and Table 1). Additionally, the review also discusses the interaction between microbiome and immunotherapy in the treatment of GB.

**TABLE 1:** Treatment approach (clinical trials) of glioblastoma.

Clinical trial	Intervention	Trial phase/total participants	Results	Remarks
*Checkpoint inhibitors*				
NCT02017717	Nivolumab vs. bevacizumab (recurrent GB)	3/530		January 3, 2023 (anticipated date to complete the study)
NCT02617589	Nivolumab + RT vs. RT + TMZ in MGMT unmethylated primary GB	3/560	13.4 vs. 13.88 months	Active, not recruiting at the moment
NCT02667587	Nivolumab + RT‐TMZ vs. RT + TMZ in MGMT methylated primary GB	3/716		Active, not recruiting at the moment
NCT02336165	Durvalumab (MEDI4736) in primary and recurrent GB	2/159	No results posted	
NCT02054806	Pembrolizumab (recurrent GB)	2/26	No results posted	
NCT02337491	Pembrolizumab alone; pembrolizumab + bevacizumab (recurrent GB)	2/80	No results posted	
NCT02054806	Pembrolizumab in advanced solid tumors	1b/26	Overall response rate was 8% (95% CI, 1%–26%)	At 6 months, the progression‐free survival rate (median, 2.8 months; 95% CI, 1.9–8.1 months) was 37.7%, and overall survival (median, 13.1 months; 95% CI, 8.0–26.6 months) was 58%.
NCT2313272	Hypofractionated stereotactic RT + pembrolizumab + bevacizumab (recurrent HGG)	1/32	No results posted	
*Vaccine*				
NCT04015700	Personalized neoantigen DNA vaccine e (GNOS‐PV01) Plasmid encoded IL‐12 (INO‐9012)	12/1	No results posted	
NCT01480479	Rindopepimut + TMZ in primary EGFRvIII positive patients	3/745	No difference in OS (2.1 vs. 20.0 month) and PFS (8.0 vs. 7.4 months)	In patients with residual tumors, there is a slight advantage.
NCT00045968	DCvax‐L in primary GB following resection	3/348		There is no clear evidence of a beneficial impact.
NCT01280552	Double‐blind, randomized study of ICT‐107 with maintenance TMZ in primary GB	2/124	There was no change in OS or PFS in the therapy arm.	Immunologically, patients in the HLA‐A2 subgroup had higher ICT‐107 activity, associated with a better clinical result.
NCT03018288	Double‐blind, randomized study of RT + TMZ and pembrolizumab+/HSPPC‐96 vaccine in primary GB	2/90		Active, not recruiting at the moment
NCT02287428	Personalized neoantigen cancer vaccine (neoVax) + RT + pembrolizumab in primary GB	2/56		Recruiting at the moment
NCT02287428	Personalized neoantigen cancer vaccine (neoVax) + RT in primary GB	1b/8	Neoantigen selection is feasible and induces an immune response	
NCT02149225	GAPVAC1 and 2, GM‐CSF and poly‐ICLC and TMZ in primary GB	1/16	Capable of eliciting a robust and long‐lasting immunological response	Unmutated APVAC1 antigens evoked long‐lasting responses from CD8+ T lymphocytes in the central memory compartment. APVAC2 elicited CD4+ T‐helper 1 responses against anticipated neoepitopes primarily.
NCT02924038	Varlimumab (CDX‐1127) + IMA950/polyICLC in primary GB	1/14		Active, not recruiting at the moment
NCT02960230	H3.3K27M peptide vaccine in children with primary DIPG/gliomas	1/49		Recruiting at the moment
*Oncolytic viral therapies*				
NCT02414165	Toca 511 (retroviral replicating vector encoding cytosine deaminase + Toca FC (flucytosine) versus lomustine, TMZ, or bevacizumab in recurrent HGG	2 and 3/403		Terminated for lack of efficacy (sponsored decision)
NCT02197169	DNX‐2401_interferon‐gamma (IFN‐) for recurrent glioblastoma	2/37	With the inclusion of IFN, there was no benefit, and IFN was poorly tolerated.	
NCT02986178	Recombinant nonpathogenic polio‐rhinovirus chimera (PVSRIPO) in recurrent malignant glioma	2/122		Active, not recruiting at the moment
NCT01470794	Toca 511 (retroviral replicating vector encoding cytosine deaminase + Toca FC (flucytosine) in recurrent HGG	1/58	Durable complete responses were observed	
NCT1491893	Recombinant nonpathogenic polio‐rhinovirus chimera (PVSRIPO) in reccurent HGG	1/61	At 36 months, there were 21% long‐term survivors.	
NCT00805376	DNX‐2401 (conditionally replication‐competent adenovirus) +/− surgery (recurrent HGG)	1/37	Long‐term survivors reported	
NCT03896568	Ad5‐DNX‐2401 (oncolytic adenovirus) in bone marrow human mesenchymal stem cells (recurrent HGG)	1/36		Recruiting at the moment
NCT01956734	DNX‐2401 + temozolomide (recurrent GB)	1/31		Completed but no result available
NCT02026271	Ad‐RTS‐hIL‐12 with veledimex in recurrent HGG	1/38	The 20 mg V dose is recommended in phase 2.	Controlled IL‐12 in recurrent GB showed promising results, with V‐dependent and proportionate increases in IL‐12 and IFN‐g leading to immune activation, a favorable safety profile, and encouraging survival.
NCT03330197	Ad‐RTS‐hIL‐12 + veledimex in pediatric subjects with brain tumors including DIPG	1/6		Terminated, sponsor decision due to slow accrual
NCT00390299	Carcinoembryonic antigen‐expressing measles virus (MV‐CEA) (recurrent GB)	1/23	No results posted	
NCT03294486	Safety and efficacy of the oncolytic virus armed for local chemotherapy, TG6002/5‐FC, in recurrent GB	1/78		Unknown status
NCT02457845	HSV G207 (oncolytic HSV‐1) + RT; children with recurrent HGG	1/12	G207 modified immunologically cool cancers into immunologically hot malignancies	In patients with recurrent or progressive pediatric high‐grade glioma, intratumoral G207 alone or in combination with radiotherapy exhibited a tolerable adverse‐event profile with evidence of responses.
NCT03152318	rQNestin34.5v0.2 (oncolytic HSV‐1) + cyclophosphamide (recurrent HGG)	1/108	CAN‐3110 was well tolerated.	Histological and molecular investigations revealed that CAN‐3110 injection was correlated to immunological activation and viral antigen persistence, indicating that it had biological activity.
NCT00390299	MV‐CEA (carcinoembryonic antigen‐expressing measles virus) (recurrent GB)	1/23	Accrual completed	Following viral treatment to a resection cavity, 6/9 (67%) patients suffered a grade 3+ adverse event, compared to 5/13 (39%) patients following intratumoral administration status after stereotactic biopsy.
NCT01301430	H‐1 PV in recurrent HGG	1/18	H‐1PV is safe and tolerable. H‐1PV can pass the blood–brain/tumor barrier and has a good survival rate.	Infected tumors showed viral multiplication, microglia/macrophage activation, and cytotoxic T cell infiltration, suggesting that H‐1PV may operate as an immunogenic stimulus.
NCT03714334	DNX‐2440 conditionally replication‐competent adenovirus with OX40 ligand (T‐cell stimulator) (recurrent GB)	1/24		Recruiting at the moment
NCT02062827	M032‐HSV‐1 (second‐generation oncolytic HSV with IL‐12 (immune stimulatory) (recurrent GB)	1/36		Recruiting at the moment
*CAR‐T cell therapy*				
NCT00730613	Genetically‐modified autologous CD8+ T cell clones for recurrent/refractory malignant glioma	1/3	Grade 3 adverse events were observed, median OS 11 months	
NCT04077866	B7‐H3‐targeted CAR T cells with or without temozolomide	1 or 2/40	No results reported	
NCT00331526	Cellular adoptive immunotherapy (recurrent GB)	2/33		Patients who obtained higher numbers of CD3+/CD16+/CD56+ (T‐LAK) cells in the cell products had a better prognosis, linked to not taking corticosteroids in the month preceding leukapheresis.
NCT04385173	B7‐H3‐targeted CAR T cells with temozolomide	12/1		Ongoing study, estimated completion date 6/2024
NCT04661384	IL13Rα2‐CAR T cells for the treatment of leptomeningeal glioblastoma, ependymoma, or medulloblastoma	1/30		Estimated primary completion date November 17, 2025
NCT2208362	IL13Rα2 CAR T cells in recurrent HGG	1/92	Estimated primary completion date June 18, 2023	Active but not recruiting at the moment
NCT02209376	EGFRvIII CAR T cells in EGFRvIII positive GBs	1/11		Terminated to pursue combination therapies
NCT01109095	HER2 virus‐specific CAR T cells	1/16		It is safe to use HER2 CAR CMV bispecific T cells. In 38% of patients, there was a long‐term clinical benefit.
NCT02442297	HER2 CAR T cells	1/28		Recruiting at the moment
*Combined treatment*				
NCT02798406	DNX‐2401 (Delta‐24‐RGD adenovirus) + pembrolizumab (Anti‐PD‐1 antibody) to trigger immune virus effects in recurrent GB	2/49		Completed but no result available
NCT02866747	Hypofractionated stereotactic radiation therapy_durvalumab (recurrent GB)	1 and 2/112	Recurrent GB, combining three 8 Gy fractions of hFSRT with 1500 mg Durvalumab on the third fraction of hFSRT and every 4 weeks is well tolerated.	Recruiting at the moment
NCT02960230	H3.3K27M peptide vaccine + nivolumab in children with primary DIPG/gliomas	1 and 2/49		Recruiting at the moment
NCT02311582	Pembrolizumab + MRI‐guided laser Ablation (recurrent malignant gliomas)	1 and 2/58		Active, not recruiting at the moment
NCT01205334	CMV‐specific cytotoxic T cells (recurrent GB)			Terminated (poor accrual)
NCT02052648	IDO inhibitor + temozolomide in recurrent HGG	1 and 2/160		Indoximod is an immunometabolic adjuvant that stimulates the activation of T cells in cancer patients.
NCT02526017	Cabiralizumab in combination with nivolumab in patients with selected advanced cancers	1/295		Cabiralizumab is a humanized monoclonal antibody that targets the colony‐stimulating factor 1 receptor, a tyrosine kinase receptor
NCT02648633	Stereotactic radiosurgery with nivolumab and valproate in patients (recurrent GB)	1/4		Terminated as nivolumab was no longer available from pharmaceutical company
NCT01811992	Combined cytotoxic and immune‐stimulatory therapy for GB	1/19	The infiltration of inflammatory cells increased in tumor samples after first resection and first recurrence, and the experiment therapy was well tolerated.	The ability of the host's brain immune system to identify gliomas has been reprogrammed, revealing a novel therapeutic strategy for highly aggressive brain tumors.

## 2 IMMUNOTHERAPEUTIC APPROACH IN GB

### 2.1 Immune checkpoints inhibitors

Immune checkpoint blockade's efficacy in GB immunotherapy has been one of the most significant breakthroughs. We examine the crucial clinical studies showing the effectiveness of monoclonal antibodies that target the proteins CTLA‐4 and PD‐1, also known as the programmed cell death protein 1 (PD‐1). These inhibitors have produced astounding outcomes, improving GB patients' quality of life and overall survival. We address potential combination strategies to improve therapy response as well as the underlying mechanisms of action.

The immune system is tasked with the crucial function of identifying and eradicating any abnormal cells, such as cancer cells. In cancer, this process is achieved through a mechanism known as immune‐mediated cytotoxicity. However, cancer cells have adapted to avoid recognition by the immune system through a phenomenon known as immune checkpoints. These checkpoints regulate the immune responses and limit excessive stimulation, preventing damage to healthy tissue. Unfortunately, in the context of GB, cancer cells can exploit these checkpoints, causing T‐cell inactivity and tolerance to the presence of the cancer cells. To overcome this challenge, researchers have developed a new therapeutic intervention, immune checkpoint inhibitors, which use monoclonal antibodies that limit T cell activation by targeting the specific surface receptors, that is, immune checkpoints to restore the immune system's ability to recognize and attack cancer cells.

Immune checkpoint inhibitors that abnormally activate immune checkpoints boost the immunotherapeutic efficacy by reducing tumor immune response and restoring T cell function. Currently, the two primary immune checkpoints are programmed cell death protein 1 (PD‐1) and cytotoxic T‐lymphocyte‐associated protein 4 (CTLA‐4).^3^ Immune checkpoint Inhibitors, specifically anti‐PD‐1 and anti‐PD‐L1 antibodies, have shown improvement in patient outcomes for cancers such as melanoma, lung cancer, and renal cancer.

Immune checkpoint inhibitors, such as ipilimumab, an antibody that inactivates CTLA‐4, have been developed to target immunosuppressive proteins, ultimately preventing and reversing immune cell exhaustion and energy. Ipilimumab was the first checkpoint blockade approved by the FDA for melanoma.^2^, ^3^ It has also been shown to have a potent effect on different brain metastases. Additionally, six other immune checkpoint inhibitors have been approved for different types of cancer, such as non‐small cell lung cancer, renal cell carcinoma, Hodgkin lymphoma, head, and neck squamous cell carcinoma, and urothelial carcinoma. These include programmed death‐1 (PD‐1) inhibitors (nivolumab, pembrolizumab, cemiplimab) and programmed death ligand‐1 (PD‐L1) inhibitors (atezolizumab, avelumab, and durvalumab).^6^

The interaction between PD‐1 and PD‐L1/2 results in the suppression of the apoptosis mechanism of tumor cells, cell exhaustion of peripheral T effector, and conversion of T effector cells to regulatory T cells (Treg).^7^, ^8^ Blocking all possible pathways downstream of PD‐1 leads to T cell anti‐tumor activity or immunity, killing cancerous cells. PD‐1 blockade is less immunotoxic than CTLA‐4 blockade, with a significantly higher recurrence‐free survival rate. A high level of PD‐L1 expression has been found in both newly diagnosed GB and recurrent GB with worse clinical outcomes.^9^ These data suggest that PD‐1 blockade is a promising strategy for treating GB patients.

The use of dual pathway blockade, CTLA‐4 blockade pathway enhancing T cell clones, Treg mediated immunosuppression, and PD‐1 blockade pathway to restore the activity of anticancer cells suggests a significant synergetic effect for an anti‐tumor immune response. This result increases the response rate in cancer patients and has been acknowledged worldwide. However, some studies have reported that the tolerability/safety of the PD‐1 checkpoint inhibitors may be superior to anti‐PD‐1 and CTLA‐4 for GB and other advanced cancer patients.^10^, ^11^, ^12^

TIM‐3 (T‐cell immunoglobulin and mucin domain‐3) and its receptor GAL‐9 (galectin‐9) are vital actors in regulating T‐cell function. When activated by GAL‐9, TIM‐3, which is predominantly expressed on worn‐out or malfunctioning T cells, induces T‐cell exhaustion, which controls immunological responses. The potential of TIM‐3/GAL‐9 inhibition as a therapeutic approach has been investigated in preclinical research and early clinical trials, especially in situations where PD‐1/PD‐L1 inhibitors have demonstrated limited success.^13^, ^14^

Another checkpoint receptor that, when activated, suppresses T‐cell proliferation and cytokine generation is LAG‐3 (lymphocyte‐activation gene 3). When T cells produce LAG‐3, it may work in concert with PD‐1 to cause a more severe form of T‐cell malfunction. In preclinical settings, combining PD‐1 inhibition with LAG‐3 blocking has demonstrated the potential to improve antitumor immune responses.^15^, ^16^

Co‐inhibitory receptors TIGIT (T cell immunoreceptor with Ig and ITIM domains) and CD96 bind to their respective ligands on APCs to adversely limit T‐cell activity.^17^ There has been interest in these checkpoints as possible targets to reduce immune suppression within the tumor microenvironment.

Investigating these alternative immune checkpoints is imperative because they might offer significant therapeutic advantages, especially when PD‐1/PD‐L1 inhibitor resistance or subpar responses occur. Furthermore, in light of the complex interactions and possible synergies among these immune checkpoints, more investigation is necessary to understand how these regulators interact and to determine the best combinatorial tactics for boosting immune responses against infectious diseases and cancer.

Clinical trials have been conducted to evaluate the efficacy and tolerability of checkpoint inhibitors in the treatment of GB. Currently, the clinical evidence for the effectiveness of immune checkpoint inhibitors is still insufficient; thus, a significant number of clinical trials are ongoing to assess the efficacy of these inhibitors (Table 1).

One such trial, CheckMate‐143 (NCT02017717), evaluated the efficiency of nivolumab, a PD‐1 inhibitor, alone or in combination with ipilimumab (a CTLA‐4 inhibitor). Results showed that nivolumab monotherapy had a greater median overall survival than the combination therapy, but the combination therapy resulted in more adverse events. In phase III of this clinical trial, the efficacy and toxicity of nivolumab (3 mg/kg) and bevacizumab (10 mg/kg) were compared in 369 patients. The patients were randomized to receive either drug every 2 weeks until confirmed disease progression. The results showed similar median overall survival and toxicity across the two groups, even though bevacizumab demonstrated a shorter period of radiologic response.^18^ An earlier phase II clinical trial assessed the effectiveness of bevacizumab alone and in combination with irinotecan, with well‐tolerated treatment arms, despite grade 3 or higher adverse events, and a median overall survival of 9.2 and 8.7 months, respectively.^19^

Two ongoing clinical trials, CheckMate‐548 (NCT02667587) and CheckMate‐498 (NCT02667587), investigate the use of nivolumab as a potential treatment for GB patients. These trials compare nivolumab in combination with temozolomide and radiation therapy to either placebo or temozolomide alone. However, phase III of a clinical trial of CheckMate 498 (NCT02617589) and phase II clinical trial of CheckMate 548 (NCT02667587) show a similar response to the overall survival time and progression‐free survival in patients with GB.^20^ The latter study is still in the preliminary phase, and additional data are required to conclude. Other clinical trials of immune checkpoints have shown promising results, including NCT02550249, NCT02337491, NCT02337686, NCT02852655, NCT03291314, NCT03047473, and NCT02336165.

A phase II trial of pembrolizumab, an anti‐PD1 checkpoint inhibitor, demonstrated increased survival in recurrent GBM patients treated with neoadjuvant pembrolizumab administration prior to surgery and post‐surgery adjuvant treatment. Other immune checkpoint receptors such as CTLA‐4, LAG‐3, and TIM‐3 have also been targeted in clinical trials for the treatment of recurrent GBM. The effects of therapies targeting CTLA‐4 (NCT02311920, NCT02829931) and LAG‐3 (NCT02658981) are still under evaluation. A phase I trial (NCT03961971) is ongoing to study the side effects of stereotactic radiosurgery with the combination of TIM‐3 inhibitor MBG453 and PD‐1 inhibitor spartalizumab.

Currently, a few clinical trials are (Phase III–NCT02617589, Phase II–NCT03925246, Phase I–NCT02852655) active but not recruiting any patients at the moment. One recent study suggests that a high dose of radiation with immune checkpoint blockade is highly effective, with a positive pathological response and enhanced survival.^21^ At the same time, another recently published article argues that CD96, a new immune checkpoint, could complement other checkpoints for a better immunotherapeutic strategy.^22^ Altogether, a single checkpoint inhibitor does not seem to produce sufficient efficacy.

Although there is great potential for using biomarkers into customized immunotherapeutic approaches for GB, several particular difficulties must be overcome. There are significant obstacles associated with the application of PD‐1/PD‐L1‐based immunotherapies. Given the variability and rare frequency of PD‐L1 expression in glioma patients, predicting treatment success requires a more sophisticated strategy. Meticulously controlled phase III trials investigating PD‐1 blocking, exemplified by nivolumab, have not been able to clearly show a survival advantage in patient cohorts with GB, despite the encouraging results seen in other cancers.^23^ One notable instance is the clinical trial CheckMate 498, where the primary survival endpoint for patients with newly diagnosed GB who received radiation therapy and PD‐1 blocking was not met.^23^ On the other hand, patients who underwent radiation and adjuvant alkylating chemotherapy with temozolomide (TMZ) had better overall survival results. These subtleties highlight the complexity of GBM and the inherent drawbacks of using PD‐1/PD‐L1‐based immunotherapeutic strategies exclusively. Therefore, the current goal should be to investigate and create alternative therapeutic procedures that can get around these limitations and maximize the effectiveness of treatment for this severe illness.

While immune checkpoint inhibitor has shown impressive results in treating a variety of cancer types, its use in GB has run into significant difficulties. In contrast to several other cancers, immune checkpoint inhibitor's effectiveness in treating GB has been considerably constrained. Unfortunately, despite having great aspirations, none of the immunotherapies—including checkpoint inhibitors—tested thus far have meaningfully improved clinical outcomes in GB patients in unselected cohorts. Due to its aggressive growth, molecular heterogeneity, and the presence of an immunosuppressive tumor microenvironment, GB is distinct and complex in many ways. To overcome these obstacles and fully utilize checkpoint inhibitor in the treatment of this deadly brain tumor, immunotherapeutic techniques for GB, including patient selection, combination therapies, and precision medicine, must be thoroughly reevaluated.

Interestingly, the dynamic interaction between immune cells and tumor cells appears to be a vital element that may increase immune checkpoint inhibitor effectiveness. In the context of immunotherapy, this complex relationship is an essential field of research. A complex ecology surrounds the tumor microenvironment, where cancer cells use a variety of tactics to avoid being seen by the immune system. Gaining knowledge about how these tumor cells interact with immune cells inside the tumor and in the surrounding tissue could lead to developing new tactics for boosting the body's reaction to immune checkpoint inhibition. These discoveries can provide light on immune evasion and resistance processes, which can lead to the development of more potent treatment strategies that interfere with these crosstalk mechanisms and, eventually, enhance the results of immune checkpoint inhibitors.

On the other hand, patients who underwent radiation and adjuvant alkylating chemotherapy with temozolomide (TMZ) had better overall survival results.^24^ These subtleties highlight the complexity of GB and the inherent drawbacks of using PD‐1/PD‐L1‐based immunotherapeutic strategies exclusively. Therefore, the current goal is to investigate and create alternative therapeutic procedures that can get around these limitations and maximize the effectiveness of treatment for this severe illness.

Altogether, a clear picture of the efficacy and tolerability of immune checkpoint inhibitors still has not been identified; thus, there is a need for a different approach to treat GB to observe the alternate result, and for that, a therapeutic synergism with a checkpoint inhibitor could be the fundamental approach to enhance overall survival time by minimizing adverse events in GB patients. Combining combination therapies with a checkpoint inhibitor may lead to better results in treating GB patients.

### 2.2 Vaccine

In recent years, vaccinations have gained significant attention as a treatment strategy against GB. Vaccination relies on the induction of an active immune response against tumor antigens, thus strengthening the adaptive immune system. Currently, there are three main approaches in the vaccine‐based treatment of GB: peptide, cell, and mRNA.

Peptide or DNA vaccines involve the injection of tumor‐specific antigens or DNA to elicit an adaptive immune response. Cell vaccines, also referred to as DC vaccines, utilize DCs derived from peripheral blood mononuclear cells and primed with tumor antigens. mRNA vaccines involve the utilization of viral vectors loaded with mRNA coding for tumor antigens, which can elicit potent immune responses.

Recent advancements in clinical research have led to increased interest in developing anti‐tumor vaccines for GB, thus resulting in several clinical trials to treat GB patients. These trials are being conducted at various stages (Table 1). These studies aim to evaluate the safety and efficacy of different vaccine candidates, with the ultimate goal of finding a treatment that can provide a good prognosis to the patients of GB. Despite the ongoing efforts in the development of vaccine‐based treatments for GB, only a few vaccination agents have reached phase III clinical trials: Rindopepimut, DCvax, and PPV. These findings underline the promise of vaccine‐based therapies as a compelling choice for GB patients and emphasize the necessity of more studies in this area.

#### 2.2.1 Peptide vaccine

The concept of using peptide vaccines in cancer immunotherapy, which involves administering small pieces of protein called peptides against specific tumor‐associated antigens to stimulate an immune response against cancer, is not new, but their application in the treatment of GB has only recently been explored. There are limitations to this approach that must be taken into consideration. For instance, tailored vaccination and genetic screening may be necessary to achieve long‐term efficacy due to the wide variation of mutations in cancer cells. Additionally, only a small number of peptide vaccines have been tested thus far, and the results of these studies have been inconclusive.

In GB, one of the most promising targets for peptide vaccination, Rindopepimut, is the epidermal growth factor receptor deletion mutation (EGFRvIII). This mutation is expressed exclusively in GB, reducing the risk of toxicity to normal cells.^25^ However, the heterogeneity of GB cells in vivo may result in tumor progression if the EGFRvIII antigen is not present.^26^ The efficacy of Rindopepimut has been tested in three uncontrolled phase II clinical trials, showing better progression‐free survival and median survival compared to historical controls.^27^ A double‐blind phase III clinical trial found no significant difference in overall survival between patients receiving Rindopepimut and those receiving a control treatment. Despite initial promising results in early clinical trials, a Phase III trial of one of the EGFRvIII‐based vaccines, Rindopepimut, was terminated after a pre‐planned interim analysis due to lack of efficacy.^28^ Another double‐blind phase III clinical trial found no significant difference in overall survival between patients receiving Rindopepimut and those receiving a control treatment.^29^

Other peptide‐based vaccines being studied for GB include IDH1 peptide and SurVaxM, which targets survivin. Early results of ongoing phase II trials for these vaccines show improved progression‐free survival and overall survival compared to historical controls.^30^ A phase I trial (NCT02507583) for an antisense oligodeoxynucleotide against insulin‐like growth factor type I receptor (IMV‐001) showed improved progression‐free survival.^31^ However, a phase III trial for a personalized protein vaccine (PPV) showed no significant improvement in overall survival compared to best supportive care.^32^

The limitations of peptide‐based vaccination in GB may be due to antigenic diversity, immune tolerance, tumor‐associated suppression, insufficient T cell activation, and so forth. In cases of recurrence of EGFRvIII‐negative tumor cells, a significant proportion of EGFRvIII expression is lost, which may explain the lack of clinical benefit observed in many Phase II and III clinical trials of peptide vaccination (Table 1).To overcome these limitations, researchers have been exploring different strategies. These strategies include modifying the peptides to enhance their immunogenicity and stability, using a combinatorial approach employing multiple peptides to target various antigens, or using the adjuvants to enhance immune response.

#### 2.2.2 Heat shock protein vaccine

Heat shock proteins (HSPs), highly conserved molecular chaperones, are essential for preserving protein homeostasis and delivering antigenic peptides to the immune system. Since HSPs are naturally processed, displayed on the cell surface, capable of inciting an immunological response against the cancerous cell. In particular, the overexpression of HSP27, HSP72, HSP73/HSP70, and HSP90 has been observed in patients with GB.

A robust immune response to GB tumors induced by HSP‐based vaccinations has been found in preclinical investigations, which is encouraging. These vaccines may be made to target several HSPs, such as HSP70 and HSP90. HSP vaccines have the ability to elicit CD4+ and CD8+ T cell‐mediated immunity by exposing the immune system to GB‐associated antigens. HSP vaccines attack cancerous cells by activating T lymphocytes, which is achieved by enhancing the uptake of antigens by APCs, thus triggering innate and adaptive immune responses against cancer cells. In addition, it has been demonstrated that the cross‐presentation of antigens by HSPs increases the effectiveness of vaccine‐induced immunity against GB.

Combining HSPs with tumor antigen peptides in a vaccine has also been shown to be effective in activating CD4+ and CD8+ T cells, which are critical players in the immune response against cancer. This approach has shown promising results in preliminary clinical trials.^33^, ^34^ Currently, two phase II clinical trials (NCT03018288 and NCT03650257) are recruiting patients with GB to test the safety and effectiveness of HSP‐peptide vaccines in combination with other treatments. These studies are an essential step toward developing new therapies for gastric cancer that harness the power of the immune system to target HSPs.

#### 2.2.3 Dendritic cell‐based vaccine

Dendritic cells are APCs that can elicit an adaptive antitumor response that can target tumors by migrating from the deep cervical lymph nodes to the tumor‐draining lymph nodes. DCs vaccines have demonstrated benefits against gliomas in preclinical models and early stages of clinical studies.

One approach to DC‐based vaccination is the use of autologous or patient‐derived, which have shown promising clinical efficacy when pulsed with tumor lysates. In a phase III study (NCT00045968) for newly diagnosed GB, the vaccine DCVax‐L showed great potential for this immunization approach when used in conjunction with standard treatment. Recently, a Phase III clinical trial of a whole tumor lysate‐pulsed DC vaccine showed positive results compared to standard therapy for newly diagnosed GB patients.^35^ The viability of mRNA‐transfected DC vaccines has been investigated in a few studies, and these vaccines may be secure and well‐tolerated. Patients with newly diagnosed GB were shown to be safe and capable of receiving tumor lysate‐pulsed DC vaccinations in addition to conventional chemoradiotherapy in a phase II clinical study.^36^

The vaccine ICT‐107, which was created especially for people with recently diagnosed GB, comprises of DCs that have been ex vivo incubated with TAAs produced in GBM cells. ICT‐107's phase I clinical study verified the safety of the vaccine. Its phase II clinical trial, which was double‐blind and placebo‐controlled, demonstrated a therapeutic effect in HLA‐A2‐positive patients.^37^, ^38^ ICT‐107 was the subject of a phase III randomized, double‐blind clinical trial; however, it had to be stopped due to limited funding.

The development of a DC‐based vaccine began around 15 years ago with ex vivo culture in mice. This approach aims to expose the patient's DCs to the glioma antigen, causing T cells to respond to the tumor cells. However, the effectiveness of this approach as an anti‐GB treatment is still unclear. Some studies have shown that RNA nanoparticles may be more effective than DCs in treating GB, while others have found that an autologous DC vaccination improves overall survival time for GB patients.^39^, ^40^, ^41^ DC‐based vaccinations for GB have been the focus of ongoing studies and discussions over their efficacy and safety. While some studies have yielded conflicting findings, others claim the vaccine is well‐tolerated and increases patient survival rates.^42^, ^43^

A study completed in 2019 found that patients who received an immunized TAA‐based cancer vaccine or tumor‐specific antigen‐based cancer vaccine had an overall survival period of 29 months, compared to 14 months for those who received standard therapy.^24^ Another study published in 2020 found that DC‐based immunization with personalized tumor‐associated antigens prompted a specific T‐cell response and led to a favorable survival outcome without any higher‐grade adverse events.^44^

A recent meta‐analysis of six randomized controlled trials found that a DC‐based vaccine significantly enhanced overall survival in newly diagnosed or recurrent GB patients.^45^ This suggests that combinatorial DC‐based vaccine therapy could be a significant advancement in the field of immunotherapy. However, before using it widely used as a routine therapy for GB, further study is required to thoroughly understand the safety and effectiveness of these vaccinations, particularly in large‐scale clinical studies.

### 2.3 Oncolytic immunovirotherapy

The field of cancer immunotherapy has rapidly expanded in recent years, with the development of new approaches aimed at harnessing the power of the immune system to fight against cancer. Among these approaches, oncolytic immunovirotherapy has emerged as a promising therapeutic strategy. By specifically targeting cancer cells while sparing healthy cells, oncolytic viruses have the potential to be highly effective in treating various forms of cancer, including GB.

The distinct strategies by which these viruses' function are key contributors to the efficacy of oncolytic immunovirotherapy. Oncolytic viruses, in particular, can inhibit the immune‐suppressing genes in tumor cells, enabling the immune system to target and eliminate them more efficiently. By transferring pro‐inflammatory and tumor genes to APCs, these viruses can also stop angiogenesis. By efficiently depriving the tumor cells of oxygen and nutrition, this process can cause their eventual death.

The potential of oncolytic viruses to draw immune cells to the tumor's location through the release of pro‐inflammatory cytokines is another crucial factor. Via a bystander effect, this immune system stimulation causes an immunological reaction that boosts the treatment's effectiveness. Oncolytic viruses may also enhance the tumor antigen encoded in the viral vector vaccine because they can provide a pro‐inflammatory immunological signal in cancer cells that can increase the production of the tumor antigen by the immune system.

Recent clinical trials have demonstrated the therapeutic potential of oncolytic immunovirotherapy for the treatment of GB and other cancer types. In one such trial, a genetically modified herpes simplex virus was shown to be effective in treating GB patients, leading to an overall survival rate of 13.6 months compared to 7.1 months in the control group.^46^ These promising results demonstrate the potential of oncolytic immunovirotherapy as a new and effective treatment option for GB patients.

Recent studies have shown that oncolytic viruses, such as adenoviruses that cause measles and herpes simplex, can significantly enhance the survival rate of GB patients. For example, a clinical trial of the oncolytic virus T‐VEC showed a significant improvement in overall survival and objective response rate in melanoma patients with advanced‐stage disease, suggesting that this approach could be effective in other types of cancer, including GB.^47^, ^48^

Moreover, a recent animal study has suggested that a low dose of Delta24‐RGD oncolytic adenovirus can upregulate PD‐1 expression on CD8+ T cells, leading to a more effective anti‐tumor response.^49^ This result is particularly intriguing since it shows that oncolytic viruses can target and eliminate cancer cells in cooperation with the immune system. Oncolytic viruses have the ability to circumvent immune suppression systems that are frequently present in tumor cells, improving the effectiveness of therapy.

However, it is essential to note that the use of oncolytic viruses in cancer treatment is still in its early stages, and several limitations must be considered. One such limitation is the host immune system's ability to mitigate viruses before they may manifest their effect. This issue can be overcome by modifying the virus's genetic material or through the use of immunosuppressive agents that can momentarily suppress the immune system's response to the virus. Oncolytic virotherapy is further limited by the emergence of viral resistance over time. Tumor cells can adapt and build defenses against the impacts of oncolytic viruses, just like they do with conventional cancer therapies. Hence, it is essential to keep developing and testing novel viral vectors in order to stay ahead of these resistance mechanisms and ensure that oncolytic virotherapy is still a viable therapeutic option. It's also crucial to choose the appropriate viral vector and delivery strategy for each form of cancer. However, the effectiveness of these therapies must be balanced against the toxicity of the used viruses and any associated adverse effects.

### 2.4 CAR T‐cell therapy

Using genetically modified antigen‐recognizing receptors in T cells (CAR‐T cells) is a promising approach to overcome the immunosuppressive GB microenvironment. Studies have shown that these cells can be highly effective in treating metastatic melanoma and other types of cancer. One of the significant benefits of CAR‐T cells is their ability to specifically target cancer antigens that other bodily cells may not be able to recognize, which can help to improve treatment outcomes and reduce the risk of side effects. For instance, treatments of T‐cells with a high dose of IL‐2 have been found to have a favorable clinical outcome in cancer patients.^50^

In addition to their ability to target specific cancer antigens, CAR‐T cells also have the potential to be more effective than other immunotherapeutic approaches based on antibodies. This is due in part to their ability to migrate through blood vessels and penetrate tumors, as well as their ability to decrease antigen expression in tumor cells while amplifying the immune response.^51^

The results of Phase I clinical trials, in which 10 patients with recurrent GB were treated with a single dose of autologous T cells directed to EGFRvIII, suggest that this approach is feasible and safe without producing off‐tumor toxicity and cytokine syndrome.^51^ However, no objective response was observed through the infusion‐targeted activity of the brain without an adaptive change in the tumor microenvironment. Furthermore, increased and robust upregulation of immune inhibitory molecules was illustrated.

In another Phase I clinical trial, chimeric antigen receptor‐engineered T cells were administered into the resected tumor cavity and ventricular system of recurrent multifocal GB patients for over 220 days targeting tumor‐associated antigen‐13 receptor alpha 2.^52^ As a result, the regression of local disease and distant metastasis (spinal metastasis) with improved quality of life was observed. This type of adoptive immunotherapy tends to be encouraging in GB patients because it can surpass the complicated tumor microenvironment of GB and the immunosuppressive stage, although the exact mechanism is unknown. Two additional Phase II clinical trials, NCT03412877 and NCT04102436, are currently recruiting patients using this treatment approach. However, while the potential benefits of CAR‐T cell therapy are clear, much more research is needed to understand the safety and efficacy of this approach entirely.

There are several different obstacles in developing a safe and efficient CAR‐T cell therapy for treating cancers of the CNS. Immune cells' limited ability to enter the CNS is the most significant of these barriers. The endothelial and epithelial blood–brain barriers are two formidable barriers that are mostly responsible for this tremendous obstacle. The intricate regulation of immune cells and chemicals entering the CNS parenchyma by these highly selective physiological barriers makes the conventional systemic administration of CAR‐T cells difficult. It is imperative that CAR‐T cells be engineered with particular characteristics to overcome these obstacles without damaging healthy brain tissue or causing harmful inflammation.

The heterogeneity of the tumor microenvironment within the CNS adds an additional layer of complexity. For example, gliomas show considerable variation in extracellular matrix composition, stromal components, and immune cell infiltration. Due to this variety, CAR‐T cell therapy responses may become inconsistent and uneven, necessitating the development of specialized techniques to account for the distinctive characteristics of each CNS tumor. A comprehensive grasp of the complex interactions among CAR‐T cells, the tumor microenvironment, and the CNS barriers is required to overcome these obstacles. Only then can novel and focused strategies be developed to overcome these obstacles and guarantee CAR‐T therapy's safe and efficient delivery to the CNS, thereby improving the prognosis of patients suffering from these incurable brain tumors.

Despite the promising results of early studies, it is essential to note that only a limited number of small chimeric antigen receptor studies have been completed to assess the safety and viability of adoptively transferred T cells. As such, it will be essential to continue conducting clinical trials to evaluate the safety and efficacy of CAR‐T cell therapy in different patient populations and with different types of cancer. Future directions should focus on CAR design optimization, enhancing T‐cell infiltration, and targeting numerous antigens for increased durability.

## 3 MICROBIOME AND IMMUNOTHERAPY INTERACTION IN GB

Immunotherapy has emerged as a promising approach for treating GB; however, the interaction between the immune system and the tumor microenvironment, which includes the microbiome, is crucial for the efficacy of immunotherapy. The human microbiome comprises a diverse ecology of bacteria, viruses, fungus, and other microbes. It contributes to several physiological functions, including metabolism, immunity, and neurobehavioral function, which are all essential to maintaining human health. However, changes in the microbiome can potentially trigger the onset of several diseases, including cancer. Recent studies suggest that gut microbiota play a crucial role in modulating the body's immune response to cancer by influencing both innate and adaptive immunity.^53^

The microbiome has been demonstrated to significantly influence tumor development and therapeutic response in GB. According to numerous research, the microbiome of GB patients differs from that of healthy people. For instance, opportunistic microorganisms like Fusobacterium nucleatum and Enterobacteriaceae, known to support tumor growth and immune evasion, have been abundant in GB patients' oral and gut microbiomes.^54^, ^55^ On the other hand, commensal bacteria, such Bifidobacterium and Lactobacillus, have been proven to improve the effectiveness of immunotherapy by stimulating immune cells and anti‐tumor responses.^56^

In addition, research has suggested that the gut microbiome may influence the response to checkpoint inhibitors, a type of immunotherapy. For example, antibiotic‐induced suppression of gut bacteria has been shown to reduce the response to immunotherapy, and certain bacterial species have been found to be associated with the effectiveness of different immunotherapy treatments.^57^ Additionally, the gut microbiome has been found to regulate the response to immunotherapy in patients with certain types of cancer, such as non‐small cell lung, kidney cancer, and melanoma.

Recent studies suggest that the gut microbiome may be critical in modulating the response to GB immunotherapy. Manipulation of the gut microbiome may be a promising strategy to improve the therapeutic response of patients who do not respond to traditional therapies. For example, some studies have shown that the presence of certain Bacteroides species, such as *B. thetaiotaomicron* and *B. fragilis*, in the gut microbiome is necessary for the effectiveness of CTLA‐4 blockade, a type of immunotherapy.^58^, ^59^

However, the relationship between the gut microbiome and GB immunotherapy is complex, and the underlying mechanisms are not yet fully understood. Further research is necessary to identify specific bacterial species or groups of bacteria that mediate anti‐tumor effects and to investigate how they interact with the human body. Advances in this field may lead to the development of new strategies for regulating the gut microbiome to stimulate anti‐tumor immune responses and improve outcomes for GB patients.

## 4 CONCLUSION AND FUTURE DIRECTION

Immunotherapy has emerged as a promising approach for treating GB, a highly aggressive and lethal brain tumor. However, the mono‐immunotherapeutic treatment approach has been shown to be insufficient in achieving conclusive clinical results. The lack of attention to the appropriate timing and sequencing of immunotherapy in conjunction with surgery and chemoradiation may be a factor contributing to this outcome. Studies have attempted to use a combination treatment approach to elicit a synergistic effect, but the desired clinical outcome has yet to be observed in GB patients.

Despite these challenges, advances in biomarker and imaging techniques offer potential solutions for assessing the effectiveness of drug delivery and the prognostic effects of treatment. Future research efforts will center on a number of crucial gaps and hurdles in advancing immunotherapy for GB. The varied character of GB necessitates a move toward customized treatment modalities, which calls for the discovery of biomarkers that can forecast the responses of certain patients to immunotherapy. Furthermore, finding ways to enhance the penetration of immunotherapeutic agents through the blood–brain barrier while maintaining safety is crucial for improving treatment efficacy. In light of these challenges, the future of GB immunotherapy research should prioritize the development of strategies that surmount these obstacles and ultimately enhance patient outcomes. A greater understanding of the GB tumor microenvironment and how immunotherapy interacts with it is crucial for developing more effective and minimally toxic treatment strategies. In addition, the advancements in genomic profiling might help in guiding treatment decisions.

Interleukin‐7 (IL‐7) agonists have the potential to counteract the lymphopenia frequently seen in GB patients, revitalizing immune function through the stimulation of T cell growth and proliferation. Including cytokine‐based treatments, such as IL‐7 agonists, in GB regimens not only addresses therapy‐induced lymphopenia but also enhances the overall effectiveness of immunotherapeutic approaches. This is a significant breakthrough in the fight against GB and presents the possibility of improving patient outcomes by utilizing the immunomodulatory power of cytokines.

In conclusion, immunotherapy has made remarkable progress in GB treatment. A rigorous and rationally designed combinatorial treatment approach, incorporating immunotherapy with optimal timing and sequencing in conjunction with other treatments, could provide unprecedented opportunities to improve patient outcomes. Future research should focus on developing more effective immunotherapy strategies for GB patients, considering the complex interplay between the immune system and the tumor microenvironment. Integrating immunotherapeutic approaches into multimodal treatment strategies holds great promise for future advancements to improve the patient's overall survival and quality of life.

## AUTHOR CONTRIBUTIONS

**Sagun Tiwari:** Conceptualization (lead); data curation (lead); formal analysis (lead); investigation (lead); resources (lead); software (lead); supervision (lead); writing – original draft (lead); writing – review and editing (lead). **Zhenxiang Han:** Conceptualization (lead); methodology (lead); project administration (lead); validation (lead); writing – original draft (lead).

## CONFLICT OF INTEREST STATEMENT

The authors have stated explicitly that there are no conflicts of interest in connection with this article.

## ETHICS STATEMENT

Not applicable.

## Data Availability

Data sharing is not applicable to this article as no new data were created or analyzed in this study.

## References

[1] Hanif F , Muzaffar K , Perveen K , Malhi SM , Simjee SU . Glioblastoma multiforme: a review of its epidemiology and pathogenesis through clinical presentation and treatment. Asian Pac J Cancer Prev. 2017;18(1):3‐9. 28239999 10.22034/APJCP.2017.18.1.3 PMC5563115

[2] Knisely JP , Yu JB , Flanigan J , Sznol M , Kluger HM , Chiang VL . Radiosurgery for melanoma brain metastases in the ipilimumab era and the possibility of longer survival. J Neurosurg. 2012;117(2):227‐233. 22702482 10.3171/2012.5.JNS111929 PMC6098938

[3] Callahan MK , Wolchok JD . At the bedside: CTLA‐4‐ and PD‐1‐blocking antibodies in cancer immunotherapy. J Leukoc Biol. 2013;94(1):41‐53. 23667165 10.1189/jlb.1212631 PMC4051187

[4] Magee DE , Hird AE , Klaassen Z , et al. Adverse event profile for immunotherapy agents compared with chemotherapy in solid organ tumors: a systematic review and meta‐analysis of randomized clinical trials. Ann Oncol. 2020;31(1):50‐60. 31912796 10.1016/j.annonc.2019.10.008

[5] Tiwari S , Sapkota N , Han Z . Effect of fasting on cancer: a narrative review of scientific evidence. Cancer Sci. 2022;113(10):3291‐3302. 35848874 10.1111/cas.15492 PMC9530862

[6] Vaddepally RK , Kharel P , Pandey R , Garje R , Chandra AB . Review of indications of FDA‐approved immune checkpoint inhibitors per NCCN guidelines with the level of evidence. Cancers (Basel). 2020;12(3):738. 10.3390/cancers12030738 PMC7140028 32245016

[7] Francisco LM , Salinas VH , Brown KE , et al. PD‐L1 regulates the development, maintenance, and function of induced regulatory T cells. J Exp Med. 2009;206(13):3015‐3029. 20008522 10.1084/jem.20090847 PMC2806460

[8] Amarnath S , Mangus CW , Wang JC , et al. The PDL1‐PD1 axis converts human TH1 cells into regulatory T cells. Sci Transl Med. 2011;3(111):111ra20. 10.1126/scitranslmed.3003130 PMC3235958 22133721

[9] Nduom EK , Wei J , Yaghi NK , et al. PD‐L1 expression and prognostic impact in glioblastoma. Neuro Oncol. 2016;18(2):195‐205. 26323609 10.1093/neuonc/nov172 PMC4724183

[10] Omuro A , Vlahovic G , Lim M , et al. Nivolumab with or without ipilimumab in patients with recurrent glioblastoma: results from exploratory phase I cohorts of CheckMate 143. Neuro Oncol. 2018;20(5):674‐686. 29106665 10.1093/neuonc/nox208 PMC5892140

[11] Wolchok JD , Kluger H , Callahan MK , et al. Nivolumab plus ipilimumab in advanced melanoma. N Engl J Med. 2013;369(2):122‐133. 23724867 10.1056/NEJMoa1302369 PMC5698004

[12] Larkin J , Chiarion‐Sileni V , Gonzalez R , et al. Combined nivolumab and ipilimumab or monotherapy in untreated melanoma. N Engl J Med. 2015;373(1):23‐34. 26027431 10.1056/NEJMoa1504030 PMC5698905

[13] Kandel S , Adhikary P , Li G , Cheng K . The TIM3/Gal9 signaling pathway: an emerging target for cancer immunotherapy. Cancer Lett. 2021;510:67‐78. 33895262 10.1016/j.canlet.2021.04.011 PMC8168453

[14] Yang R , Sun L , Li CF , et al. Galectin‐9 interacts with PD‐1 and TIM‐3 to regulate T cell death and is a target for cancer immunotherapy. Nat Commun. 2021;12(1):832. 33547304 10.1038/s41467-021-21099-2 PMC7864927

[15] Gestermann N , Saugy D , Martignier C , et al. LAG‐3 and PD‐1+LAG‐3 inhibition promote anti‐tumor immune responses in human autologous melanoma/T cell co‐cultures. Onco Targets Ther. 2020;9(1):1736792. 10.1080/2162402X.2020.1736792 PMC7422827 32850194

[16] Robert C . LAG‐3 and PD‐1 blockade raises the bar for melanoma. Nature Cancer. 2021;2(12):1251‐1253. 35121906 10.1038/s43018-021-00276-8

[17] Dougall WC , Kurtulus S , Smyth MJ , Anderson AC . TIGIT and CD96: new checkpoint receptor targets for cancer immunotherapy. Immunol Rev. 2017;276(1):112‐120. 28258695 10.1111/imr.12518

[18] Reardon DA , Brandes AA , Omuro A , et al. Effect of nivolumab vs bevacizumab in patients with recurrent glioblastoma: the CheckMate 143 phase 3 randomized clinical trial. JAMA Oncol. 2020;6(7):1003‐1010. 32437507 10.1001/jamaoncol.2020.1024 PMC7243167

[19] Friedman HS , Prados MD , Wen PY , et al. Bevacizumab alone and in combination with irinotecan in recurrent glioblastoma. J Clin Oncol. 2009;27(28):4733‐4740. 19720927 10.1200/JCO.2008.19.8721

[20] Woroniecka K , Fecci PE . Immuno‐synergy? Neoantigen vaccines and checkpoint blockade in glioblastoma. Neuro Oncol. 2020;22(9):1233‐1234. 32691060 10.1093/neuonc/noaa170 PMC7523436

[21] Brown NF , Ng SM , Brooks C , et al. A phase II open label, randomised study of ipilimumab with temozolomide versus temozolomide alone after surgery and chemoradiotherapy in patients with recently diagnosed glioblastoma: the Ipi‐Glio trial protocol. BMC Cancer. 2020;20(1):198. 32164579 10.1186/s12885-020-6624-y PMC7068928

[22] Liu F , Huang J , He F , et al. CD96, a new immune checkpoint, correlates with immune profile and clinical outcome of glioma. Sci Rep. 2020;10(1):10768. 32612110 10.1038/s41598-020-66806-z PMC7330044

[23] Omuro A , Brandes AA , Carpentier AF , et al. Radiotherapy combined with nivolumab or temozolomide for newly diagnosed glioblastoma with unmethylated MGMT promoter: an international randomized phase III trial. Neuro Oncol. 2023;25(1):123‐134. 35419607 10.1093/neuonc/noac099 PMC9825306

[24] Stupp R , Mason WP , van den Bent MJ , et al. Radiotherapy plus concomitant and adjuvant temozolomide for glioblastoma. N Engl J Med. 2005;352(10):987‐996. 15758009 10.1056/NEJMoa043330

[25] Weller M , Kaulich K , Hentschel B , et al. Assessment and prognostic significance of the epidermal growth factor receptor vIII mutation in glioblastoma patients treated with concurrent and adjuvant temozolomide radiochemotherapy. Int J Cancer. 2014;134(10):2437‐2447. 24614983 10.1002/ijc.28576

[26] Felsberg J , Hentschel B , Kaulich K , et al. Epidermal growth factor receptor variant III (EGFRvIII) positivity in EGFR‐amplified glioblastomas: prognostic role and comparison between primary and recurrent tumors. Clin Cancer Res. 2017;23(22):6846‐6855. 28855349 10.1158/1078-0432.CCR-17-0890

[27] Yuan B , Wang G , Tang X , Tong A , Zhou L . Immunotherapy of glioblastoma: recent advances and future prospects. Hum Vaccin Immunother. 2022;18(5):2055417. 35344682 10.1080/21645515.2022.2055417 PMC9248956

[28] Malkki H . Trial watch: glioblastoma vaccine therapy disappointment in phase III trial. Nat Rev Neurol. 2016;12(4):190. 10.1038/nrneurol.2016.38 27020557

[29] Weller M , Butowski N , Tran DD , et al. Rindopepimut with temozolomide for patients with newly diagnosed, EGFRvIII‐expressing glioblastoma (ACT IV): a randomised, double‐blind, international phase 3 trial. Lancet Oncol. 2017;18(10):1373‐1385. 28844499 10.1016/S1470-2045(17)30517-X

[30] Ahluwalia MS , Reardon DA , Abad AP , et al. Phase IIa study of SurVaxM plus adjuvant temozolomide for newly diagnosed glioblastoma. J Clin Oncol. 2023;41(7):1453‐1465. 36521103 10.1200/JCO.22.00996 PMC9995096

[31] Andrews DW , Judy KD , Scott CB , et al. Phase Ib clinical trial of IGV‐001 for patients with newly diagnosed glioblastoma. Clin Cancer Res. 2021;27(7):1912‐1922. 33500356 10.1158/1078-0432.CCR-20-3805

[32] Narita Y , Arakawa Y , Yamasaki F , et al. A randomized, double‐blind, phase III trial of personalized peptide vaccination for recurrent glioblastoma. Neuro Oncol. 2019;21(3):348‐359. 30500939 10.1093/neuonc/noy200 PMC6380422

[33] Bloch O , Lim M , Sughrue ME , et al. Autologous heat shock protein peptide vaccination for newly diagnosed glioblastoma: impact of peripheral PD‐L1 expression on response to therapy. Clin Cancer Res. 2017;23(14):3575‐3584. 28193626 10.1158/1078-0432.CCR-16-1369 PMC5511566

[34] Ji N , Zhang Y , Liu Y , et al. Heat shock protein peptide complex‐96 vaccination for newly diagnosed glioblastoma: a phase I, single‐arm trial. JCI Insight. 2018;3(10):e99145. 10.1172/jci.insight.99145 PMC6012501 29769450

[35] Liau LM , Ashkan K , Tran DD , et al. First results on survival from a large phase 3 clinical trial of an autologous dendritic cell vaccine in newly diagnosed glioblastoma. J Transl Med. 2018;16(1):142. 29843811 10.1186/s12967-018-1507-6 PMC5975654

[36] Inogés S , Tejada S , de Cerio AL , et al. A phase II trial of autologous dendritic cell vaccination and radiochemotherapy following fluorescence‐guided surgery in newly diagnosed glioblastoma patients. J Transl Med. 2017;15(1):104. 28499389 10.1186/s12967-017-1202-z PMC5427614

[37] Wen PY , Reardon DA , Armstrong TS , et al. A randomized double‐blind placebo‐controlled phase II trial of dendritic cell vaccine ICT‐107 in newly diagnosed patients with glioblastoma. Clin Cancer Res. 2019;25(19):5799‐5807. 31320597 10.1158/1078-0432.CCR-19-0261 PMC8132111

[38] Phuphanich S , Wheeler CJ , Rudnick JD , et al. Phase I trial of a multi‐epitope‐pulsed dendritic cell vaccine for patients with newly diagnosed glioblastoma. Cancer Immunol Immunother. 2013;62(1):125‐135. 22847020 10.1007/s00262-012-1319-0 PMC3541928

[39] Sayour EJ , De Leon G , Pham C , et al. Systemic activation of antigen‐presenting cells via RNA‐loaded nanoparticles. Onco Targets Ther. 2017;6(1):e1256527. 10.1080/2162402X.2016.1256527 PMC5283636 28197373

[40] Bregy A , Wong TM , Shah AH , Goldberg JM , Komotar RJ . Active immunotherapy using dendritic cells in the treatment of glioblastoma multiforme. Cancer Treat Rev. 2013;39(8):891‐907. 23790634 10.1016/j.ctrv.2013.05.007

[41] Mitchell DA , Batich KA , Gunn MD , et al. Tetanus toxoid and CCL3 improve dendritic cell vaccines in mice and glioblastoma patients. Nature. 2015;519(7543):366‐369. 25762141 10.1038/nature14320 PMC4510871

[42] Buchroithner J , Erhart F , Pichler J , et al. Audencel immunotherapy based on dendritic cells has no effect on overall and progression‐free survival in newly diagnosed glioblastoma: a phase II randomized trial. Cancers (Basel). 2018;10(10):372. 10.3390/cancers10100372 PMC6210090 30301187

[43] Srivastava S , Jackson C , Kim T , Choi J , Lim M . A characterization of dendritic cells and their role in immunotherapy in glioblastoma: from preclinical studies to clinical trials. Cancers (Basel). 2019;11(4):537. 10.3390/cancers11040537 PMC6521200 30991681

[44] Wang QT , Nie Y , Sun SN , et al. Tumor‐associated antigen‐based personalized dendritic cell vaccine in solid tumor patients. Cancer Immunol Immunother. 2020;69(7):1375‐1387. 32078016 10.1007/s00262-020-02496-w PMC11027674

[45] Lv L , Huang J , Xi H , Zhou X . Efficacy and safety of dendritic cell vaccines for patients with glioblastoma: a meta‐analysis of randomized controlled trials. Int Immunopharmacol. 2020;83:106336. 32213460 10.1016/j.intimp.2020.106336

[46] Cloughesy TF , Landolfi J , Hogan DJ , et al. Phase 1 trial of vocimagene amiretrorepvec and 5‐fluorocytosine for recurrent high‐grade glioma. Sci Transl Med. 2016;8(341):341ra75. 10.1126/scitranslmed.aad9784 PMC6707068 27252174

[47] Andtbacka RH , Kaufman HL , Collichio F , et al. Talimogene Laherparepvec improves durable response rate in patients with advanced melanoma. J Clin Oncol. 2015;33(25):2780‐2788. 26014293 10.1200/JCO.2014.58.3377

[48] Bommareddy PK , Patel A , Hossain S , Kaufman HL . Talimogene Laherparepvec (T‐VEC) and other oncolytic viruses for the treatment of melanoma. Am J Clin Dermatol. 2017;18(1):1‐15. 27988837 10.1007/s40257-016-0238-9 PMC8977104

[49] Belcaid Z , Berrevoets C , Choi J , et al. Low‐dose oncolytic adenovirus therapy overcomes tumor‐induced immune suppression and sensitizes intracranial gliomas to anti‐PD‐1 therapy. Neurooncol Adv. 2020;2(1):011. 10.1093/noajnl/vdaa011 PMC7212906 32642679

[50] Rosenberg SA , Yang JC , Sherry RM , et al. Durable complete responses in heavily pretreated patients with metastatic melanoma using T‐cell transfer immunotherapy. Clin Cancer Res. 2011;17(13):4550‐4557. 21498393 10.1158/1078-0432.CCR-11-0116 PMC3131487

[51] O'Rourke DM , Nasrallah MP , Desai A , et al. A single dose of peripherally infused EGFRvIII‐directed CAR T cells mediates antigen loss and induces adaptive resistance in patients with recurrent glioblastoma. Sci Transl Med. 2017;9(399):eaaa0984. 10.1126/scitranslmed.aaa0984 PMC5762203 28724573

[52] Brown CE , Alizadeh D , Starr R , et al. Regression of glioblastoma after chimeric antigen receptor T‐cell therapy. N Engl J Med. 2016;375(26):2561‐2569. 28029927 10.1056/NEJMoa1610497 PMC5390684

[53] Zheng D , Liwinski T , Elinav E . Interaction between microbiota and immunity in health and disease. Cell Res. 2020;30(6):492‐506. 32433595 10.1038/s41422-020-0332-7 PMC7264227

[54] Lin B , Ye Z , Ye Z , et al. Gut microbiota in brain tumors: an emerging crucial player. CNS Neurosci Ther. 2023;29:84‐97. 36627748 10.1111/cns.14081 PMC10314108

[55] Knippel RJ , Drewes JL , Sears CL . The cancer microbiome: recent highlights and knowledge gaps. Cancer Discov. 2021;11(10):2378‐2395. 34400408 10.1158/2159-8290.CD-21-0324 PMC8487941

[56] Bae J , Park K , Kim YM . Commensal microbiota and cancer immunotherapy: harnessing commensal bacteria for cancer therapy. Immune Netw. 2022;22(1):e3. 35291651 10.4110/in.2022.22.e3 PMC8901697

[57] Wu J , Wang S , Zheng B , Qiu X , Wang H , Chen L . Modulation of gut microbiota to enhance effect of checkpoint inhibitor immunotherapy. Front Immunol. 2021;12:669150. 34267748 10.3389/fimmu.2021.669150 PMC8276067

[58] Vétizou M , Pitt JM , Daillère R , et al. Anticancer immunotherapy by CTLA‐4 blockade relies on the gut microbiota. Science. 2015;350(6264):1079‐1084. 26541610 10.1126/science.aad1329 PMC4721659

[59] Miller PL , Carson TL . Mechanisms and microbial influences on CTLA‐4 and PD‐1‐based immunotherapy in the treatment of cancer: a narrative review. Gut Pathog. 2020;12:43. 32944086 10.1186/s13099-020-00381-6 PMC7488430

